# Pediatric Staphylococcal Scalded Skin Syndrome Complicated by Methicillin-Resistant Staphylococcus Aureus and Steroid Abuse: A Case Report

**DOI:** 10.31729/jnma.8770

**Published:** 2024-10-31

**Authors:** Ujjwal Kumar Jha, Ajay Kumar Yadav

**Affiliations:** 1 Grande International Hospital, Dhapasi, Kathmandu, Nepal; 2 Patan Academy of Health Sciences, Lagankhel, Lalitpur, Nepal

**Keywords:** *case report*, *MRSA*, *skin*, *Staphylococcal*

## Abstract

Staphylococcal Scalded Skin Syndrome (SSSS) is a severe exfoliative skin condition primarily affecting children, caused by the toxins produced by *Staphylococcus aureus.* This case report describes the clinical presentation, diagnostic challenges, and successful management of SSSS complicated by Methicillin-Resistant *Staphylococcus aureus* (MRSA) in a pediatric patient.

A pediatric patient presented with a two-week history of pruritic vesiculobullous eruptions that progressively worsened. Despite initial treatments with various topical and oral medications including steroid and antibiotic ointments, the lesions continued to progress. A wound culture identified heavy growth of MRSA, prompting a change in the treatment regimen, resulting in significant clinical improvement.

This case underscores the importance of accurate diagnosis and timely intervention in the management of SSSS, particularly when complicated by MRSA. The successful outcome with appropriate antibiotic therapy highlights the need for a multidisciplinary approach and emphasizes the value of culture and sensitivity testing in guiding effective treatment strategies.

## INTRODUCTION

Staphylococcal Scalded Skin Syndrome (SSSS) is characterized by skin denudation caused by exotoxin-producing Staphylococcus strains and is rare in children over six.^1^ Two exfoliative toxins A and B cause red rashes and epidermal separation, forming bullae and diffuse desquamation.^2^ Diagnosis is clinical, supported by the presence of *Staphylococcus aureus* in the cultural exams.^3^ If untreated, SSSS can cause severe morbidity and mortality.^4^ Very few cases have been reported involving SSSS with Methicillin-Resistant *Staphylococcus aureus* (MRSA) infection and a history of steroid abuse. We report a case of a 2-year-old female with SSSS.

## CASE REPORT

**Figure 1:**
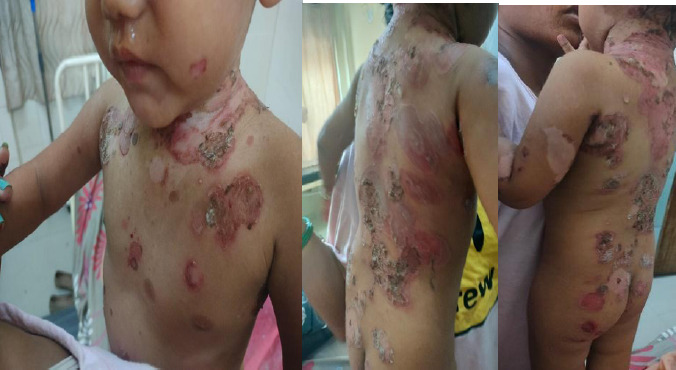
Superficial erythematous rashes, ruptured bullae and diffuse desquamation of skin over neck, axilla and back

A 2-year female presented in the dermatology outpatient department (OPD) with chief complaints of pruritic, painful vesiculobullous eruptions over the generalized body for 2 weeks. The onset was acute, with gradual progression. Initially, the eruptions began as pruritic vesicles over the lateral aspect of the left arm, later becoming bullous and spontaneously rupturing. The patient also experienced fever at the onset of eruptions, which was continuous with a maximum temperature of 100°F, not associated with chills and rigors, and relieved with paracetamol. The eruptions gradually spread to involve the back, bilateral gluteal region, bilateral lower limbs, face, and scalp (Figure 1).

The birth history revealed that the pregnancy and delivery were unremarkable. The child was born at term via spontaneous vaginal delivery. Child was fully immunized as per the expanded programme on immunization schedule of Nepal.

The patient was initially managed with oral medications syrup (chlorphenaramine + paracetamol) and topical application of (Beclometasone 0.025% w/w + Neomycin 0.5% w/w + Clotrimazole 1% w/w) cream on recommendation of local pharmacy, which did not provide relief. Subsequently, the patient was managed with a combination of (Betamethasone 0.5% w/w + Clotrimazole 1% w/w + Gentamicin 0.1% w/w) ointment, Acyclovir 5% w/w ointment, oral Cloxacillin 100 mg TDS and oral Ampicillin 125 mg TDS. Despite these interventions, the lesions continued to progress and patient was brought to Patan Hospital.

Upon examination, the patient was found to be active, feeding well, and afebrile. Multiple areas of yellowish-white crusted plaques were observed over the upper chest, left lower leg, right forehead, nape of the neck, and back. Additionally, multiple eroded bullae with surrounding erythema were noted over the back, along with a single large flaccid bullae containing straw-colored fluid. Few vesicles were present over the left arm and pubic region.

Given the worsening condition and history of ineffective treatment, pus swab from wound site was taken and sent for gram stain, culture and antibiotic sensitivity. Patient was admitted to the pediatric ward under the dermatology unit. The initial management included syrup Cefadroxil 125 mg twice a day, syrup Paracetamol 125 mg twice a day, ointment mupirocin and Condy's compress for wound care.

Swab culture revealed heavy growth of Methicillin-Resistant *Staphylococcus aureus* (MRSA). Antibiotic sensitivity test is shown in Table 1.

**Table 1:** Antibiotic sensitivity test

Resistance	Sensitive
Oxacillin	Vancomycin
Erythiromycin	Chloramphenicol
Clindamycin	Doxycycline
Ofloxacin	Linezolide
Ciprofloxacin	
Trimethoprim - Sufamethoxazole	
Gentamicin	
Azithromycin	

In laboratory findings, complete blood count and biochemistry were within normal limits. After reviewing the antibiotic sensitivity test report, the patient was commenced on a 7-day course of intravenous Vancomycin and Chloramphenicol. The patient remained hemodynamically and clinically stable throughout the hospitalization period, with no new eruptions observed at the time of discharge.

## DISCUSSION

Staphylococcal Scalded Skin Syndrome (SSSS) is a severe exfoliative skin condition primarily affecting children, caused by the exfoliative toxins produced by *Staphylococcus aureus*. In this case, the patient presented with a history of pruritic vesiculobullous eruptions that progressively worsened over two weeks, despite initial treatments with various topical and oral medications. This progression is consistent with typical SSSS cases, where initial vesicles rapidly evolve into larger bullae that rupture easily.^5,2^

The patient's clinical examination revealed multiple yellowish-white crusted plaques and eroded bullae with surrounding erythema as typical SSSS presentations.^6^ The identification of MRSA in the wound culture underscores the importance of precise microbial identification and antibiotic sensitivity testing in the management of SSSS, where rapid progression and septic complications were observed.^7^

The initial treatments, including the use of chlorpheniramine, paracetamol, and various topical antibiotics and topical steroid use, were ineffective, which is a common challenge noted in managing SSSS. Corticosteroid therapy suppresses inflammation, but it can also encourage the growth of fungi or bacteria.^8^ When using topical steroids in neonates and small children, extra care is necessary as they tend to absorb more medication applied topically because of their higher ratio of total body surface area to body weight.^8^

Insufficient awareness regarding the proper use and potential side effects of topical steroids can have severe, even catastrophic consequences. Enforcing stricter laws to limit over-the-counter dispensing of medications could help prevent worsening of disease. The successful response to this regimen, coupled with supportive care, mirrors the positive outcomes reported in other case studies when appropriate antibiotic therapy is administered promptly.^2^
